# Nonenzymatic Browning of Amorphous Maltose/Whey Protein Isolates Matrix: Effects of Water Sorption and Molecular Mobility

**DOI:** 10.3390/foods11142128

**Published:** 2022-07-18

**Authors:** Yaowen Wu, Haoxuan Ye, Fanghui Fan

**Affiliations:** 1 Department of Food Science and Engineering, College of Chemistry and Environmental Engineering, Shenzhen University, Shenzhen 518060, China; wuyaowen2019@email.szu.edu.cn (Y.W.); yehaoxuan2021@email.szu.edu.cn (H.Y.); 2 Institute of Advanced Study, Shenzhen University, Shenzhen 518060, China; 3 Shenzhen Key Laboratory of Food Macromolecules Science and Processing, Shenzhen University, Shenzhen 518060, China

**Keywords:** nonenzymatic browning, water sorption, reaction kinetics, strength analysis, molecular mobility, maltose

## Abstract

Nonenzymatic browning (NEB) reactions often affect the nutritional quality and safety properties of amorphous food solids. Developing a proper approach to control the NEB reaction has been of particular interest in the food industry. An NEB reaction in an amorphous maltose/Whey protein isolates (WPI) matrix containing L-lysine and D-xylose as reactants were studied at ambient temperatures *a_w_* ≤ 0.44 and 45~65 °C. The results indicated that the presence of NEB reactants barely disturbed the water sorption behavior of the matrix. The Guggenheim–Anderson–de Boer (GAB) constants and *Q_st_* values of the studied samples were affected by storage conditions as the migration of sorbed water among monolayers occurred. The rate of color changes and 5-hydoxymethylfurfural (5-HMF) accumulation on the matrix were accelerated at high ambient temperatures *a_w_*, reflecting the extent of NEB reaction increases. Since the strength concept (*S*) could give a measure of molecular mobility, the extent of the NEB reaction was governed by the molecular mobility of the matrix as the activation energy (*E_a_*) of 5-HMF production minimized at solids with high S values. We found that the *S* concept had a considerable potential usage in controlling the NEB reaction on amorphous sugar–protein solids. This data set has practical significance in the comprehensive understanding of manipulating the diffusion-limited chemical reactions on low-moisture food solids.

## 1. Introduction

NEB, including the Maillard reaction, as a model of diffusion-limited reaction, is one of the most crucial chemical phenomena in the food thermal process [1,2,3]. The NEB reaction often takes place between α-amino groups (proteins, peptides, or amino acids) and reducing sugars (carbonyl group), which can generate high molecular weight compounds called melanoidins [4]. Furthermore, contributing to food palatability by improving flavors and colors, it is important to know that the NEB reaction can also be unfavorable in foods [5,6]. For instance, the browning generations can affect the nutritional quality and safety properties of food products as toxicological conjugates such as acrylamide produce in the reaction [7,8]. Therefore, developing a proper approach to control the rate of NEB reaction has been of particular interest in recent years for the food processing industry and nutritional realms.

The diffusion-controlled chemical reactions are mainly dependent on the translational diffusivity of the reactants (or on the viscosity of the matrix), and thus, are susceptible to the physical state of the matrix [9]. Previous studies have reported that the extent of NEB reaction in food solids can be optimized through careful manipulation of the phase and state transitions of matrix materials such as crystallization and glass transition [10,11]. Glass transition may result internally in food solids because of changes in plasticizer after water sorption, which can dramatically change the molecular diffusion of the matrix [3]. Despite water plasticization, heat liberates because water molecules are at a lower energy state on the adsorbent surface than in atmosphere when the water sorption takes place [12]. The above heat transfer is one of the key variables to determine the water content and the structural transformation of the solid matrix [13]. Since the kinetics of water sorption and molecular diffusion on food solids rapidly change around *T_g_* of the matrix, the combination of water sorption characteristics and glass transition can offer an innovative approach in manipulating diffusion-controlled chemical developments in food solids. However, few studies have reported the applicability of such information in controlling NEB reaction on food solids.

Since the NEB reaction is highly dependent on the molecular collision between reactants, the water sorption-induced molecular mobility itself may directly impact its reactivity in low and intermediate moisture systems [14]. This occurred because the probability of collision is restricted by the low molecular mobility of the glassy matrix [15,16]. On the contrary, a rapid increase in collision occurs when temperature increases above the *T_g_* of the matrix, as the molecular mobility is accelerated by absorbed water [17,18,19]. The molecular mobility of the amorphous sugar/protein matrix could be observed and measured by several techniques, e.g., DMA [18], DEA [20], and ^1^H NMR [21]. To quantify the molecular mobility, *S* has been introduced to describe the molecular mobility associating *T_g_* with the food solids composed of carbohydrates, proteins, and other components [11,16,22]. The *S* concept, describing a viscose flow of molecules in glass, has a greater bearing on the thermal and mechanical behavior as relevant to applications in the handling and transportation of solids. Furthermore, when *S*, identifying an allowable temperature range, increases above *T_g_*, it intuitively expresses the temporal–spatial response-ability of molecules to change in motion [16]. Since the relationship between the molecular mobility of the matrix and NEB reaction has not been studied much, we expect the *S* concept to be considered as using information for controlling NEB reactivity in a systematic manner to guide the thermal process of sugar-rich solid foods.

The quality and stability of sugar-rich foods are often governed by the physicochemical properties of sugars [9,10]. As an essential starch derivative, maltose [4-(α-D-Glucopyranosido)-α-glucopyranose] has already become a common additive in many sugar-rich foods [23]. Moreover, the WPI, as polymeric food components, may act as stabilizers, which are widely used in food structure formulation [16]. Our previous studies point out that the WPI can enhance the molecular mobility of maltose and increase the S value of the maltose-containing food models [23]. To understand the effect of molecular mobility on chemical reactions, in this paper, maltose and WPI were chosen as research targets. Meanwhile, the *S* value of the above sugar–protein matrix is calculated to investigate the relationship between molecular mobility and the NEB reactivity in the studied matrix. The NEB reaction not only contributes to food palatability by improving flavors and colors but affects the nutritional quality and safety properties of products in the food industry. Hence, controlling the reaction rate is necessary for food processing and safety guarantees. This paper can provide a comprehensive understanding of manipulating diffusion-limited NEB reactions in sugar–protein food models, which has practical significance in improving the processability and stability of sugar-rich foods.

## 2. Materials and Methods

### 2.1. Sample Preparation

D-(+)-maltose monohydrate (Sigma-Aldrich, St. Louis, MO, USA) and WPI (Mullins Whey Inc., Mosinee, WI, USA; including carbohydrates or lipids as impurities <10%) were used to compose an amorphous sugar–protein matrix in this paper. Previous studies pointed out that the NEB reactivity was highly related to the protein structure changes or to a broken glycosidic linkage of a sugar–protein solid matrix [24,25]. In this study, however, the storage temperature range was not high enough to denature the structure of WPI. Furthermore, the hydrolysis of glycosidic linkage could barely occur in amorphous maltose at low water activities (≤0.44 *a_w_*). Therefore, the NEB reactivity on the maltose/WPI solid matrix would be extremely low at studied conditions, which was in agreement with previous reports [1,10]. To investigate the relationship between molecular mobility and NEB reaction rates, therefore, the xylose and lysine were added as the NEB reactants because of their high NEB reactivity [15]. Specifically, maltose and WPI solutions (20% in total solids, *w*/*w*) were prepared separately in deionized water at 25 °C and subsequently mixed to obtain maltose/WPI solutions with solid ratios of 7:3, 1:1, and 3:7 by mass. The xylose and lysine (in the mass ratio of 1:1) were adjusted to 5% (*w*/*w*) of the total solids in the prepared maltose/WPI solutions [15]. Further, 5 mL portions of the solutions, loaded in preweighed 20 mL glass vials (semiclosed), were frozen in a still-air freezer (DW-HL240, Zhongkemeiling Co., Ltd., Hefei, China) at −20 °C for 24 h. They were subsequently tempered at −80 °C for 3 h before freeze-drying (10N/B, Scientz, Ningbo, China) until the chamber pressure was below 2 bar. Three units of each sample were stored in vacuum desiccators (Chuxi Industrial Co. Ltd., Shanghai, China) over desiccant (P_2_O_5_, Sigma-Aldrich, St. Louis, MO, USA) to avoid water sorption and reach equilibrium at room temperature.

### 2.2. Water Sorption Testing

Freeze-dried samples were stored in vacuum desiccators over the saturated solutions of LiCl, CH_3_COOK, MgCl_2_, and K_2_CO_3_ for 0.13, 0.22, 0.33, and 0.44 *a_w_* (Sigma-Aldrich, St. Louis, MO, USA) to avoid crystallization. All the vacuum desiccators were stored in incubators (IMH750-S, ThermoFisher, Massachusetts, MA, USA), setting temperatures at 45, 55, and 65 °C, respectively. During storage, the samples were weighed for 12 h until the weights leveled off. The Guggenheim–Anderson–de Boer (GAB) model (Equation (1)) was utilized, where *m* and *m*_0_ referred to the weighted water content and monolayer water content. Moreover, the *Q_st_* (kJ∙mol^−1^) gave a measure of enthalpy changes when water adsorbed from the atmosphere to the surface of solids [12,26]. The *Q_st_* was determined from water sorption data using the Clausius–Clapeyron equation (Equation (2)), where *R* was the universal gas constant (8.314 J∙mol^−1^K^−1^) and *a_w_*_1_ and *a_w_*_2_ were water activity at different experimental temperatures *T*_1_ and *T*_2_ [27]. It should be noted that the *a_w_*_1_ and *T*_1_ in Equation (2) were chosen as 0.11 and 25 °C to compare with the previous studies [28].

(1) $$\frac{m}{m_{0}}=\frac{C_{GAB}K_{GAB}a_{w}}{\left(1-K_{GAB}a_{w}\right)\left(1-K_{GAB}a_{w}+C_{G}K_{GAB}a_{w}\right)}$$

(2) $$\ln(\frac{a_{w1}}{a_{w2}})=-\frac{Q_{st}}{R}\left(\frac{1}{T_{1}}-\frac{1}{T_{2}}\right)$$

### 2.3. Color Changes Measurement

The color changes of the studied samples were measured using a portable colorimeter (CR-400, Konica-Minolta, Tokyo, Japan). Daily calibration was implemented by using the white ceramic tile for reference. Samples were filled in a vial and measured directly without any application. The color changes usually represented in the L^*^, a^*^ and b^*^ color space in the food industry [29]. Similarly, in this study, the L^*^ value corresponding to the luminance or lightness component (lightness or brightness; 0 = black, 100 = white) was recorded as the sign of NEB reaction main depending on luminance [30]. Moreover, the Δ*E* were calculated from the initial and final values of L^*^, a^*^ and b^*^ of each formulation by Equation (3) for evaluating the extent of the NEB reaction [31].

(3) $$\Delta E=\sqrt{\Delta {a*}^{2}+\Delta {b*}^{2}+\Delta {L*}^{2}}$$

### 2.4. HPLC Analysis

Numerous studies had reported that the 5-hydoxymethylfurfural (5-HMF), as an intermediate product via NEB reaction, is an indicator for determining the severity of NEB reaction in thermally treated foods [32]. Therefore, the accumulation of 5-HMF concentration in prepared samples was quantified using a HPLC (U3000, ThermoFisher, Massachusetts, MA, USA) at the following settings: Chromeleon software (version 7.2.1, Thermo-Scientific Co., Waltham, MA, USA), C30 column (5 μm,4.6 mm × 250 mm; ThermoFisher, Massachusetts, MA, USA), and column temperature at 30 °C. Absorption peaks measured in both 280 nm and 420 nm as yellow and brown pigments were derived. The product of 5-HMF in the prepared samples was monitored for 12 h for up to 5 days during storage. The integrated peak areas derived from different storage times were used for evaluating the production of 5-HMF [30].

### 2.5. NEB Reaction Kinetics

Previous studies reported that the production of 5-HMF was the rate-limited step for NEB reactions [33]. In this study, the production of 5-HMF derived from HPLC was fitted by a zero-order reaction model (Equation (4)), where *C_H_* was the concentration at time t (day), *C_H_*_0_ was the concentration at time zero, and *k* was the rate constant (the rate of 5-HMF production: *k_H_*). Since the kinetic-related *E_a_* represented the minimum amount of energy that must be provided to compounds to result in a chemical reaction, the Arrhenius equations (Equation (5)) were used to give the quantitative basis of the relationship between the *E_a_* and the kinetic information of the NEB reaction in this paper.

(4) $$C_{H}=K_{H}t+C_{H0}$$

(5) $$lnK_{H}=lnA-\frac{E_{a}}{RT}$$

In Equation (5), the *k_H_* refers to the rate constant (mg∙day^−1^), *E_a_* is the activation energy (kJ∙mol^−1^), *A* is the constant, *R* is the universal gas constant (8.314 J∙mol^−1^K^−1^), and *T* is the absolute temperature [34,35].

### 2.6. Molecular Mobility Analysis

The strength concept, as a measure of molecular mobility on food solids, was initially constructed by the William–Landel–Ferry (WLF) analysis of mechanical-/dielectric-related α-relaxation data above the overall calorimetric *T_g_* of solids [16]. It should be noted that the *S* of glass-forming sugar-rich food mixtures was mainly governed by water content as the *T_g_* and relaxation behavior was component-dependent [36]. Previous studies reported that the *S* value of noncrystalline sugar–protein mixtures could be predicted in Equation (6), including water content, calorimetric onset-*T_g_*, and *S* value for each component at a dry state [36]. Our previous study had reported a relationship including dispersed phases and relaxation processes of amorphous maltose, which showed that the *S* data of anhydrous maltose was 20.1 °C [23]. In this paper, the *S* values for amorphous maltose/WPI mixtures can be calculated, as the corresponding water contents of each noncrystalline component were estimated by the GAB model based on Equation (1) at the studied *a_w_* and temperature.

(6) $$S_{p}=\frac{w_{1}S_{d1}+k_{s}w_{2}S_{d2}}{w_{1}+k_{s}w_{2}}$$

where the *S_p_* refers to the predicted value of mixtures, *w*_1_ and *w*_2_, respectively, refer to the mass fractions of dry solid and water, *k_s_* is a water content proportional constant, and *S_d_* refers to the experimental *S* value for anhydrous sugar and water (*S_d_*_1_ = 20.1 °C for anhydrous maltose and *S_d_*_2_ = 1.3 °C for pure water sourcing from literature data [23]).

### 2.7. Statistical Analysis

The water sorption isotherms, color values, and HPLC data of triplicate measurements were plotted in Microsoft Excel (2019, Microsoft, Inc., Albuquerque, NM, USA). The average values with a standard deviation of triplicate measurements were calculated. Additionally, the error bars and significance analysis were implemented in the confidence interval of 95% to represent the variability of data.

## 3. Results

### 3.1. Water Sorption Isotherms

**Figure 1:**
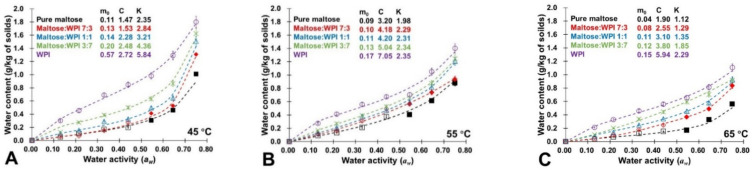
The experimental water sorption (empty points, 0.11~0.44 *a_w_*) and calculated water content data (solid points, 0.56~0.76 *a_w_*) for the amorphous maltose/WPI mixtures at the studied storage conditions. The GAB-derived monolayer value (*m*_0_) and constants *C_GAB_* and *K_GAB_* for each studied sample were calculated and shown. (**A**) Water sorption data derived from 45 °C; (**B**) Water sorption data derived from 55 °C; (**C**) Water sorption data derived from 65 °C.

The experimental data of water sorption for all mass ratios of amorphous mixtures under various conditions (0~0.44 *a_w_* and 45~65 °C) are shown in Figure 1 and fitted by the GAB model. Although most of the studied samples achieved steady states after 24 h of storage, the equilibrium sorption data for the amorphous maltose/WPI mixtures were achieved at *a_w_* ≤ 0.44 and 45 °C to 65 °C up to 120 h, which is in agreement with previous reports [23]. This indicated that the presence of a tiny quantity of reactants barely affected the water sorption behavior of the amorphous sugar–protein matrix. Previous studies reported that the water sorption behavior of the amorphous sugar–protein solids was a result of fractional quantities (Equation (7)) [37,38]. In this paper, similar water additive principles were observed in amorphous maltose/WPI mixtures at *a_w_* below 0.44 and the studied temperature range (45~65 °C). Therefore, in the present study, the calculated water content (steady-state sorbed water of noncrystalline components) for maltose/WPI mixtures at 0.56~0.76 *a_w_* were used in the GAB model according to Equation (7) shown in Figure 1. For example, the GAB sorption isotherm of noncrystalline maltose was obtained using experimental data (0.11~0.44 *a_w_*) and data derived from maltose/WPI mixture at 3:7 (*w*/*w*) from 0.55 to 0.76 *a_w_* based on Equation (7). Additionally, the GAB sorption isotherms for noncrystalline maltose/WPI systems at 7:3 and 1:1 (*w*/*w*) used experimental data 0.11~0.44 *a_w_* and fractional water contents for noncrystalline maltose and data measured for maltose/WPI mixture at 3:7 (*w*/*w*) to predict sorbed water contents up to 0.76 *a_w_*. Moreover, the GAB-derived monolayer value (*m*_0_) of maltose/WPI solids increased with WPI-content increases and were found to be the sum of the fractional quantities at the studied conditions. This agreed with previous studies that reported that the water additive principle in amorphous sugar–protein solids also exists at the molecular level [18,23].

*W_t_* = *n*_1_*W*_1_ +, ⋯, + *n_n_W_n_* (7)

where *W_t_* is the total equilibrium water content in the system; *n*_1_, ⋯, *n_n_* is the mass fraction of each component in the system; and *W*_1_, ⋯, *W_n_* are the water contents sorbed by each component [37,38].

The GAB constant *C_GAB_* related to the logarithmical difference of the magnitude of the chemical potential of sorbed water between the first monolayer and the upper layers, while the constant K_GAB_ was related, to a degree of freedom, to representing this difference in the sorbate’s pure liquid state and in the upper layers [28,39]. The *C_GAB_* value of the studied samples increased with ambient *a_w_* and storage temperature, whereas the *K_GAB_* values were depressed by high storage temperature at constant *a_w_* (Figure 1). This implied that the sorbed water in the upper layer more easily underwent desorption, reflecting on the *C_GAB_* increases, where less-sorbed water in the upper layer left as a smaller K_GAB_ showed at high *a_w_* and storage temperature. To evaluate hydrogen bonding among the first and upper layer of sorbed water and the surface of solids, the *Q_st_* of each studied sample were calculated (Table 1). The *Q_st_* values of amorphous pure maltose decreased with increasing *a_w_* or storage temperatures (Table 1). The high *Q_st_* at low *a_w_* was caused by the strong hydrogen bonding of water molecules to the surface of amorphous solids constituting a monolayer of molecules, and then the amount of energy required to remove these water molecules was high [27,40]. It should be noted that the *Q_st_* is governed by the extent of surface loading for sorbates on an energetically heterogeneous surface [28,41]. In this study, the presence of WPI could lower the *Q_st_* of amorphous pure maltose at a constant *a_w_* and storage temperature (Table 1). Previous studies reported that the polymeric food components, such as WPI, possessed much more water-bonding sites than those in sugars [18]. Therefore, this occurred because the extent of surface loading for water molecules was enhanced by the WPI addition. Moreover, the determination of the GAB constants and *Q_st_* values provided data for industrial energy consumption calculations and the design of drying equipment for sugar-rich foods.

**Figure 2:**
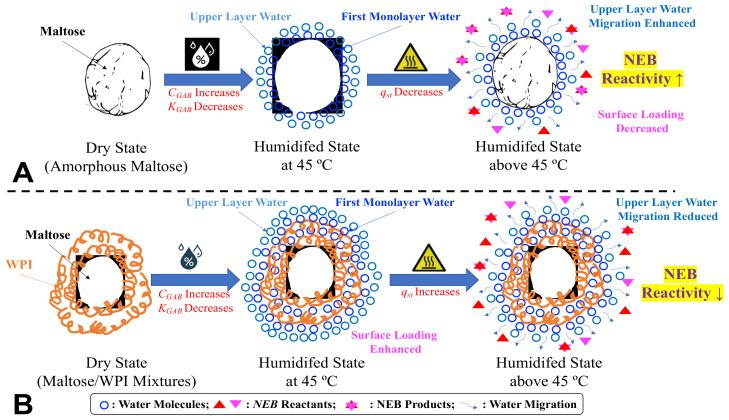
The schematic diagram of the sorbed water migration on the surface of the studied amorphous maltose/WPI matrix at molecular levels. The symbols do not represent the real sizes and quantity of components. (**A**) water migration for amorphous maltose; (**B**) water migration for amorphous maltose/WPI mixtures.

Rather than water affecting chemical reactions via *a_w_* or by plasticizing amorphous systems and considering the inhibitory effect of water as a reaction product of NEB reactions, water migration itself may directly impact chemical reactivity in low- and intermediate-moisture systems [26]. As noted above, Figure 2 demonstrates a schematic diagram to explain the water migration on the surface of amorphous maltose/WPI solids at molecular levels based on the combination of GAB constants and *Q_st_* values. For pure amorphous maltose, the upper layer of sorbed water was easier to migrate than the water sorbed at the first monolayer at high ambient *a_w_* or storage temperatures (Figure 2A). This is because the latter was more firmly bound to the water-bonding sites on the surface of solids than the former, as high interaction energies are required for desorption. Since polymeric food components could enhance the extent of surface loading of amorphous maltose, the protein addition would make it difficult to remove water, as the energy required for desorption increases (Figure 2B). Therefore, the migration of sorbed water among monolayers could impact chemical reactivity in low- and intermediate-moisture food solids.

### 3.2. Color Changes Measurement

**Figure 3:**
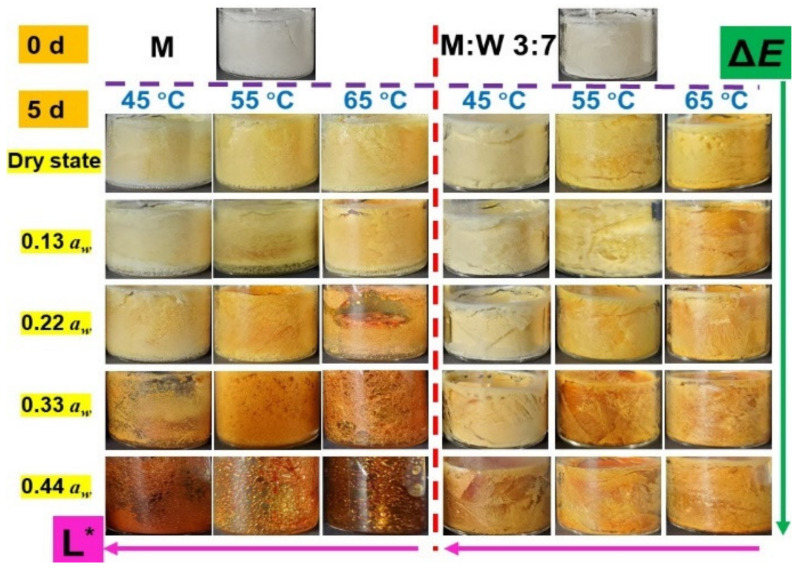
The color changes of the studied samples after storage under various conditions (0~0.44 *a_w_* and 45~65 °C) at 5 days. The pure maltose and maltose/WPI (3:7, *w*/*w*), which only experienced freeze-drying, were chosen as referencing comparations.

Figure 3 exhibits the color changes of the studied samples (pure maltose and maltose/WPI with 3:7 in mass after storage under various conditions (0~0.44 *a_w_* and 45~65 °C)) until 5 days. Compared to the referenced freeze-dried samples, the color of amorphous pure maltose extensively became brown after storage, as the NEB reaction occurred. The extent of the color changes was enhanced by the ambient *a_w_* and storage temperatures, whereas the presence of WPI could slow down the browning, especially at high a_w_ and temperature (Figure 3). Özhan and others [30] reported that the NEB reaction mainly depended on luminance and the L* value, which, according to the luminance or lightness component, could be considered as the sign. In this paper, Figure 3 shows that the L* of studied samples decreased concomitantly with *a_w_* and storage temperature. Meanwhile, the Δ*E* was calculated based on the L*, a*, and b* values of each studied sample for evaluating the extent of the NEB reaction, as shown in Table 2. It should be noted that the higher the Δ*E* in each sample, the more obvious the color changes are [31]. The Δ*E* values of amorphous maltose/WPI matrix increased with the increase of *a_w_* and storage temperature (Table 2), which indicated that the extent of the NEB reaction was accelerated by a_w_ and temperature. Since the color changes were directly related to the extent of the NEB reactions [30], the rates of color changes in prepared samples were monitored in this paper. Table 2 shows the rate of color changes (*k_C_*) of the studied samples after storage at various conditions. The *k_C_* increased with ambient *a_w_* or storage temperature increases (Table 2). In pure amorphous maltose, for example, the *k_C_* values of pure maltose stored at 0.44 *a_w_* were much higher than the dry state at any of the studied storage temperatures. Moreover, the increasing in storage temperature could rapidly increase the *k_C_* values of amorphous maltose/WPI solids, especially at high *a_w_* conditions. It should be noted that the NEB reaction was highly dependent on the molecular collision between reactants [14]. Since the *Q_st_* was depressed by high temperature as noted above, increasing storage temperature could encourage upper-layer sorbed water to migrate. These migrated water molecules probably participated in the NEB reaction by increasing the probability of molecular collision [10,42]. However, the presence of WPI could depress the extent of color changes, as the *k_C_* values of amorphous maltose/WPI matrix decreased with the WPI content increases (Table 2). This was caused because fewer water molecules were left to accelerate the NEB reaction, as the extent of surface loading was enhanced by WPI. Therefore, the above results indicated that the sorbed water acted as a main factor affecting the color changes of amorphous food solids, as the diffusion-limited chemical reactions could be accelerated by water migration.

### 3.3. HPLC Measurements

**Figure 4:**
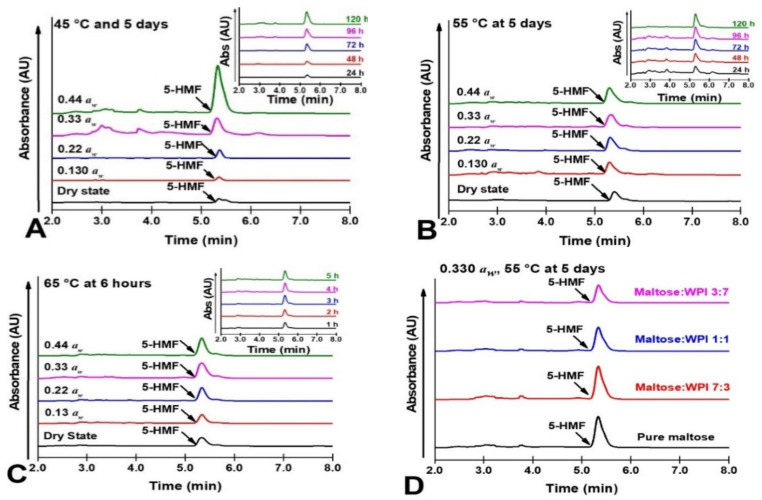
The HPLC chromatograms for amorphous maltose/WPI matrix at the studied storage conditions (dry state~0.44 *a_w_* and 45~65 °C) shown in (**A**–**D**) demonstrated the HPLC chromatograms of pure maltose and maltose/WPI mixture (7:3, 1:1, and 3:7, *w*/*w*) at 0.33 *a_w_* and 55 °C. Time-dependent curve for pure maltose (0.33 *a_w_*) is shown in (**A**–**C**). The peaks in approximately 5.3 min were identified and integrated to represent the 5-HMF accumulation [43].

The accumulation of intermediate products in the NEB reaction (5-HMF) measured using HPLC analysis in the amorphous maltose/WPI matrix at the studied storage conditions (dry state~0.44 *a_w_* and 45~65 °C) are shown in Figure 4. In this study, the chromatograms showed that the retention time of 5-HMF arises approximately after 5.3 min (Figure 4), which agreed with the previous studies. Quantitative values of 5-HMF retention were determined using the characteristic peak areas of 5-HMF [43]. The 5-HMF in amorphous maltose and maltose/WPI mixtures accumulated with ambient *a_w_* and storage temperatures (Figure 4A–C). Since the accumulation of 5-HMF was an indicator for determining the severity of NEB reaction, this result indicated that the sorbed water and storage temperature could enhance the NEB reactivity. However, the presence of WPI decreased the production of 5-HMF as the integrated peak area at 5.3 min decreased with the increase in WPI content (Figure 4B). This was probably because the water sorption behavior was changed by WPI, which then disturbed the NEB reaction. For the rate of 5-HMF accumulation based on the HPLC measurement, similarly, the *k_H_* values of the studied samples were governed by ambient *a_w_* and storage temperatures, while the WPI addition delayed the 5-HMF accumulation (Table 2). As the diffusion-controlled chemical reactions were particularly dependent on the physical state of the matrix, previous studies had reported that the extent of the NEB reaction was optimized through careful manipulation of the glass transition [9,10,11]. Our previous study reported that the *T_g_* value of dry maltose was 51.1 °C, which would be decreased by the addition of *a_w_* and increased by the addition of WPI [23]. The above phenomenon indicated that the rate of NEB reaction accelerated as the glass transition occurred. Since glass transition dramatically changed the molecular diffusion of the sugar–protein solid matrix [20,44], the water sorption-reduced state transition might change the NEB reactivity.

### 3.4. NEB Reaction Kinetics

**Figure 5:**
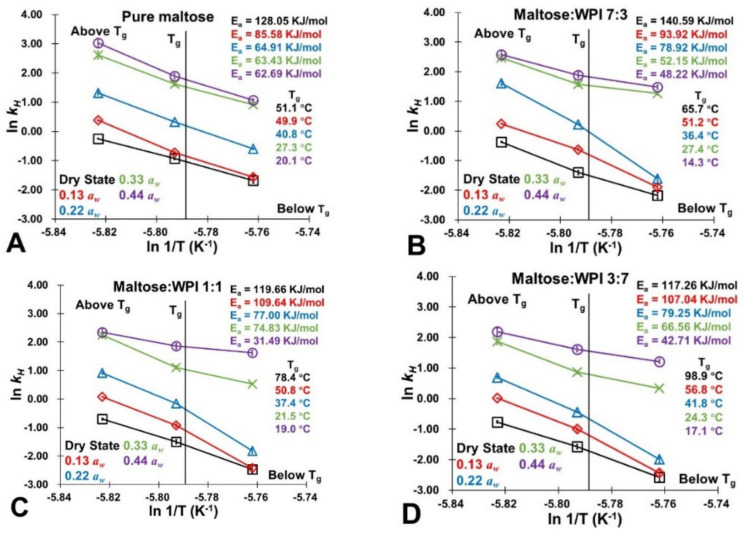
The Arrhenius plots of amorphous maltose and maltose/WPI mixtures after storage at the studied conditions (dry state~0.44 *a_w_* and 45~65 °C). The activation energies (*E_a_*) were calculated based on *k_H_* values and the literature *T_g_* value of pure maltose was shown [23]. (**A**) Pure maltose; (**B**) Maltose:WPI 7:3; (**C**) Maltose:WPI 1:1; (**D**) Maltose:WPI 3:7.

Previous studies reported that the accumulation of 5-HMF was a typical zero-order reaction as the rate of 5-HMF production was proportional to the zeroth power of reactants concentration [3,30,33]. In this study, similarly, the *k_H_* of each studied sample was obtained as straight lines were fitted in the plots of 5-HMF accumulations against storage time (5 days) with high correlation coefficients (*R*^2^ > 0.9575), which supported the application of zero order kinetics (Table 2). Similar to the rate of color changes (*k_C_*), the *k_H_* of each studied sample was governed by the physical state of the matrix as the *k_H_* values rapidly decreased at temperatures above the literature *T_g_* data of amorphous maltose (Figure 5). This was caused by a large-scale molecular motion which was induced above *T_g_*, which could enhance the NEB reactivity.

In chemical reactions, *E_a_* refers to the minimum amount of energy that must be provided to compounds to result in reactions. On the other hand, *E_a_* acted as the magnitude of the potential energy barrier separating minima of the potential energy surface pertaining to the initial and final thermodynamic state [45,46]. To evaluate the spontaneity of the NEB reaction, the Arrhenius plots of amorphous maltose and maltose/WPI mixtures after storage at the studied conditions (dry state~0.44 *a_w_* and 45~65 °C) and corresponding E_a_ were developed and are shown in Figure 5. In this study, the *E_a_* values of the studied samples decreased from a dry state to 0.44 *a_w_* at a constant storage temperature (Figure 5). This indicated that the sorbed water could minimize the potential energy barrier in the system, probably due to the water plasticization rapid molecular mobility of the matrix, and thus, could enhance the extent of NEB reaction. Nevertheless, the presence of WPI increased the *E_a_* of the studied samples (Figure 5). Furthermore, based on the effects of the surface loading trapping more water, the protein-derived physical blocking could delay the molecular mobility of the matrix as well as the NEB reactivity [47]. The relationship between molecular mobility and NEB reactivity will continue to be discussed in the next section.

### 3.5. Molecular Mobility and NEB Reactivity

**Figure 6:**
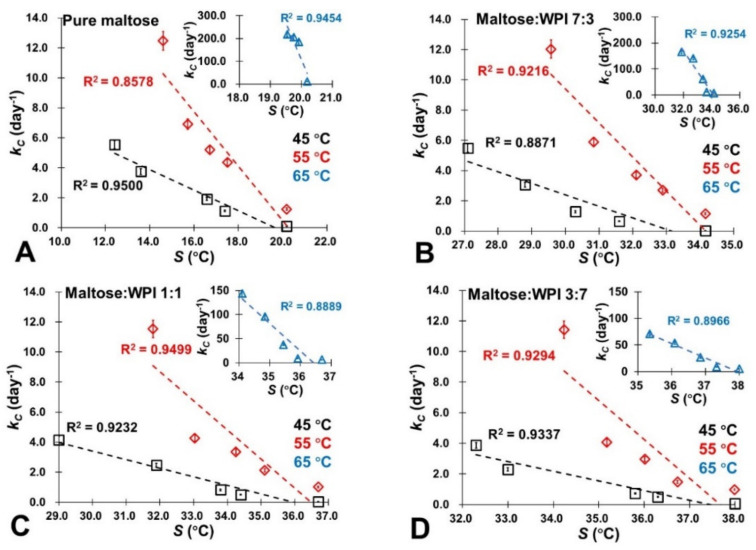
The relationship between the rate of color changes (*k_C_*) of the studied samples based on colorimetric measurement and their corresponding *S* values calculated based on the literature [23]. (**A**) Pure maltose; (**B**) Maltose:WPI 7:3; (**C**) Maltose:WPI 1:1; (**D**) Maltose:WPI 3:7.

**Figure 7:**
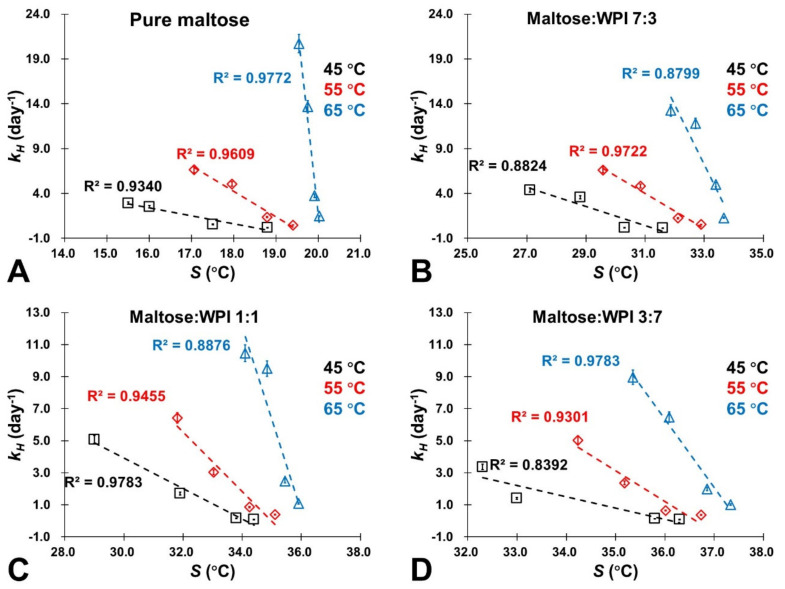
The relationship between the accumulation rate of 5-HMF (*k_H_*) of the studied samples based on the HPLC measurements and their corresponding *S* values calculated based on the literature [23]. (**A**) Pure maltose; (**B**) Maltose:WPI 7:3; (**C**) Maltose:WPI 1:1; (**D**) Maltose:WPI 3:7.

**Figure 8:**
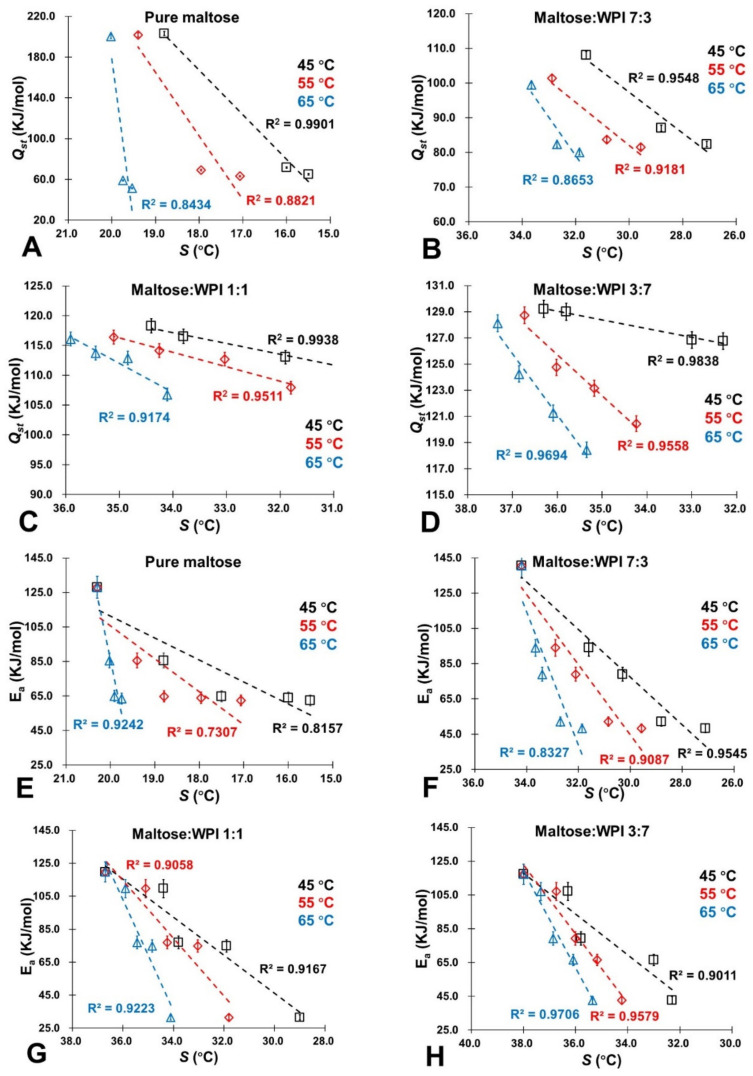
The relationship between *Q_st_* and *E_a_* associating with the S values of the studied samples at the studied storage conditions (0.11~0.76 *a_w_*; 45 °C~65 °C). (**A**) *Q_st_* for pure maltose; (**B**) *Q_st_* for maltose:WPI 7:3; (**C**) *Q_st_* for maltose:WPI 1:1; (**D**) *Q_st_* for maltose:WPI 3:7; (**E**) *E_a_* for pure maltose; (**F**) *E_a_* for maltose:WPI 7:3; (**G**) *E_a_* for maltose:WPI 1:1; (**H**) *E_a_* for maltose:WPI 3:7.

It should be noted that *a_w_* (or water content) provided an estimation of molecular mobility as its relationship with *S* in amorphous sugars was established and gave quantified measurements [22]. In this study, Table 3 provides the S values of the studied maltose/WPI matrix calculated by using the literature data [23]. It shows that the *S* value decreased with increasing water content, which indicates that the sorbed water would influence the molecular mobility of the maltose/WPI matrix. Moreover, the presence of WPI could improve the S value of amorphous sugar because the protein retarded the molecular mobility of amorphous maltose (Table 3). For the controlling of NEB reactivity, a linear relationship with a correlation coefficient (*R*^2^) of association above 0.8 was found between the rate of color changes and 5-HMF products (*k_C_* and *k_H_*) and the literature S values of the studied samples at various storage conditions (dry state ~ 0.44 *a_w_* and 45 ~ 65 °C), which is demonstrated in Figure 6 and Figure 7. Specifically, both *k_C_* and *k_H_* decreased concomitantly with the S increases, indicating that the color changes and NEB reactivity depended on the molecular mobility of the matrix. Since a small S value is referred to as a more rapid structural change in the amorphous materials [23], this resulted because sorbed water enhanced the molecular mobility in the matrix by disrupting the H-bonded networks and then accelerated the NEB reactivity. As a small-molecule-weight plasticizer, water has a significant effect on the large-scale molecular motion of food solids [48,49]. As we noted above, the *Q_st_* values represented the amount of energy required to remove the water molecules bonded to the surface of solids constituting a monolayer of molecules [27]. In this study, the relationship between the *Q_st_* value and S is shown in Figure 8A–D, where the *Q_st_* value decreased as the *S* value decreases. This was caused by the high molecular mobility of the matrix, which could minimize the sorption energy and release more water molecules from the surface of amorphous solids, intensifying the NEB reactions. Furthermore, the potential energy barrier of the NEB reaction was also minimized when the molecular mobility of the matrix increased as the *E_a_* value decreased concomitantly with decreases in the *S* values Figure 8E–H. Therefore, the *S* concept had a considerable potential usage in controlling the NEB reaction on amorphous sugar–protein solids as well as improving the nutritional quality and safety properties of food products in thermal process and storage.

## 4. Conclusions

We found that the GAB constants and *Q_st_* values of the studied samples were affected by storage conditions as the water migration among monolayers occurs. The extent of the NEB reaction could be enhanced by water sorption and glass transition. Since the S gave a measure of molecular mobility, the extent of the NEB reaction was governed by the molecular mobility of the matrix. The findings of this paper suggest an alternative approach to control the NEB reaction in high-sugar foods, which has a potential usage in many food productions, e.g., high-sugar solution spray drying, cereal dehydration, and powder handling. For further research, the relationship between the S and the diffusion-limited physical and chemical processes on complex sugar-containing food solids as well as the real food model will be continually investigated.

## Figures and Tables

**Table 1:** The *Q_st_* in pure amorphous maltose (M) and maltose/WPI (M-W) mixtures at the studied storage conditions (0.13 to 0.44 *a_w_* and 45 to 65 °C).

	M		M-W 7:3		M-W 1:1		M-W 3:7		WPI	
	Water Contents (g/kg of Solids)	*Q_st_* (kJ∙mol^−1^)	Water Contents (g/kg of Solids)	*Q_st_* (kJ∙mol^−1^)	Water Contents (g/kg of Solids)	*Q_st_* (kJ∙mol^−1^)	Water Contents (g/kg of Solids)	*Q_st_* (kJ∙mol^−1^)	Water Contents (g/kg of Solids)	*Q_st_* (kJ∙mol^−1^)
45 °C										
0.13 *a_w_*	0.05 ± 0.01 ^a^ *	203.0 ± 2.2 ^a^	0.06 ± 0.01 ^a^	108.0 ± 1.2 ^a^	0.11 ± 0.03 ^a^	118.3 ± 0.7 ^a^	0.13 ± 0.03 ^a^	129.2 ± 2.5 ^a^	0.30 ± 0.06 ^a^	124.1 ± 1.7 ^a^
0.22 *a_w_*	0.11 ± 0.03 ^b^ **	91.9 ± 1.1 ^b^	0.07 ± 0.02 ^b^	85.7 ± 0.3 ^b^	0.16 ± 0.02 ^b^	116.5 ± 1.6 ^b^	0.27 ± 0.09 ^b^	129.0 ± 0.2 ^b^	0.46 ± 0.07 ^b^	122.4 ± 1.4 ^b^
0.33 *a_w_*	0.17 ± 0.04 ^c^	71.5 ± 0.9 ^c^	0.15 ± 0.05 ^c^	87.0 ± 1.48 ^c^	0.29 ± 0.06 ^c^	113.0 ± 1.7 ^c^	0.39 ± 0.08 ^c^	126.8 ± 0.9 ^c^	0.69 ± 0.02 ^c^	115.1 ± 1.5 ^c^
0.44 *a_w_*	0.20 ± 0.02 ^d^	65.0 ± 3.4 ^d^	0.28 ± 0.05 ^d^	82.4 ± 2.2 ^d^	0.33 ± 0.07 ^d^	108.3 ± 1.4 ^d^	0.50 ± 0.13 ^d^	126.8 ± 1.2 ^d^	0.85 ± 0.08 ^d^	110.3 ± 1.7 ^d^
55 °C										
0.13 *a_w_*	0.09 ± 0.02 ^a^	201.7 ± 3.5 ^a^	0.11 ± 0.07 ^a^	101.3 ± 3.7 ^a^	0.17 ± 0.01 ^a^	116.4 ± 1.4 ^a^	0.17 ± 0.02 ^a^	128.7 ± 0.7 ^a^	0.27 ± 0.02 ^a^	114.9 ± 2.0 ^a^
0.22 *a_w_*	0.13 ± 0.01 ^b^	89.9 ± 1.9 ^b^	0.16 ± 0.02 ^b^	85.4 ± 0.8 ^b^	0.26 ± 0.03 ^b^	114.1 ± 1.2 ^b^	0.27 ± 0.05 ^b^	124.8 ± 0.5 ^b^	0.41 ± 0.04 ^b^	111.8 ± 1.5 ^b^
0.33 *a_w_*	0.21 ± 0.08 ^c^	69.2 ± 1.3 ^c^	0.31 ± 0.10 ^c^	83.7 ± 2.6 ^c^	0.43 ± 0.09 ^c^	112.7 ± 2.2 ^c^	0.39 ± 0.06 ^c^	123.2 ± 1.2 ^c^	0.56 ± 0.08 ^c^	109.9 ± 1.8 ^c^
0.44 *a_w_*	0.38 ± 0.05 ^d^	63.1 ± 1.7 ^d^	0.49 ± 0.07 ^d^	81.6 ± 1.0 ^d^	0.47 ± 0.04 ^d^	107.9 ± 1.0 ^d^	0.53 ± 0.03 ^d^	120.4 ± 1.1 ^d^	0.67 ± 0.12 ^d^	107.6 ± 1.3 ^d^
65 °C										
0.13 *a_w_*	0.04 ± 0.01 ^a^	20.25 ± 1.0 ^a^	0.04 ± 0.01 ^a^	99.5 ± 1.7 ^a^	0.08 ± 0.02 ^a^	116.1 ± 0.3 ^a^	0.11 ± 0.03 ^a^	128.1 ± 1.5 ^a^	0.21 ± 0.02 ^a^	112.4 ± 1.2 ^a^
0.22 *a_w_*	0.07 ± 0.02 ^b^	83.5 ± 1.8 ^b^	0.10 ± 0.01 ^b^	83.8 ± 1.5 ^b^	0.14 ± 0.05 ^b^	113.7 ± 1.5 ^b^	0.20 ± 0.01 ^b^	124.2 ± 2.5 ^b^	0.33 ± 0.06 ^b^	109.4 ± 0.8 ^b^
0.33 *a_w_*	0.12 ± 0.01 ^c^	59.0 ± 1.6 ^c^	0.18 ± 0.02 ^c^	81.3 ± 0.9 ^c^	0.25 ± 0.02 ^c^	112.9 ± 3.2 ^c^	0.34 ± 0.04 ^c^	121.3 ± 1.0 ^c^	0.46 ± 0.04 ^c^	109.2 ± 0.7 ^c^
0.44 *a_w_*	0.15 ± 0.02 ^d^	51.4 ± 2.2 ^d^	0.25 ± 0.03 ^d^	80.0 ± 2.5 ^d^	0.34 ± 0.01 ^d^	106.7 ± 1.9 ^d^	0.44 ± 0.08 ^d^	118.4 ± 3.4 ^d^	0.56 ± 0.02 ^d^	106.4 ± 1.7 ^d^

*: Significant analysis at *p* < 0.05; **: Values are means ± SDs (*n* = 3). Different superscript letters indicate significant differences.

**Table 2:** The rate of color changes (*k_C_*, day^−1^), color difference (Δ*E*), the rate of 5-HMF accumulations (*k_H_*, day^−1^), and *R*^2^ of zero-order reaction for the amorphous pure amorphous maltose (M) and maltose/WPI (M-W) mixtures at the studied conditions (dry state~0.44 *a_w_* and 45~65 °C).

	M				M-W 7:3				M-W 1:1				M-W 3:7			
	Δ*E*	*k_C_*	*k_H_*	*R* ^2^	Δ*E*	*k_C_*	*k_H_*	*R* ^2^	Δ*E*	*k_C_*	*k_H_*	*R* ^2^	Δ*E*	*k_C_*	*k_H_*	*R* ^2^
45 °C																
Dry	469.0 ± 1.9 ^a^ *	0.1 ± 0.2 ^a^	0.2 ± 0.0 ^a^	0.9994	313.3 ± 2.6 ^a^	0.03 ± 0.24 ^a^	0.1 ± 0.2 ^a^	0.9922	313.8 ± 3.4 ^a^	0.04 ± 0.15 ^a^	0.1 ± 0.1 ^a^	0.9979	258.8 ± 3.1 ^a^	0.1 ± 0.22 ^a^	0.1 ± 0.1 ^a^	0.9970
0.13 *a_w_*	579.6 ± 2.3 ^b^ **	1.1 ± 0.1 ^b^	0.2 ± 0.1 ^a^	0.9919	319.0 ± 1.4 ^b^	0.7 ± 0.2 ^b^	0.2 ± 0.2 ^b^	0.9904	329.1 ± 2.0 ^b^	0.5 ± 0.2 ^b^	0.1 ± 0.0 ^a^	0.9894	282.2 ± 2.2 ^b^	0.5 ± 0.2 ^b^	0.1 ± 0.0 ^b^	0.9923
0.22 *a_w_*	668.8 ± 1.4 ^c^	1.9 ± 0.3 ^c^	0.4 ± 0.1 ^b^	0.9988	349.6 ± 1.8 ^c^	1.3 ± 0.2 ^c^	0.2 ± 0.1 ^b^	0.9951	388.4 ± 2.3 ^c^	0.8 ± 0.1 ^c^	0.2 ± 0.1 ^b^	0.9867	284.6 ± 3.5 ^b^	0.7 ± 0.2 ^c^	0.1 ± 0.4 ^c^	0.9935
0.33 *a_w_*	1216.0 ± 4.3 ^d^	3.8 ± 0.1 ^d^	4.2 ± 0.4 ^b^	0.9885	579.6 ± 2.2 ^d^	3.1 ± 0.1 ^d^	3.6 ± 0.4 ^b^	0.9807	576.8 ± 1.9 ^d^	2.5 ± 0.2 ^d^	2.3 ± 0.5 ^c^	0.9626	419.5 ± 1.7 ^c^	2.3 ± 0.2 ^d^	1.7 ± 1.1 ^d^	0.9650
0.44 *a_w_*	1551.0 ± 3.0 ^e^	5.6 ± 0.1 ^e^	4.7 ± 0.9 ^d^	0.9895	1264.4 ± 3.5 ^e^	5.5 ± 0.1 ^e^	6.5 ± 0.8 ^d^	0.9757	437.3 ± 2.8 ^e^	4.2 ± 0.1 ^e^	5.1 ± 0.3 ^d^	0.9575	618.4 ± 1.9 ^d^	3.9 ± 0.1 ^e^	3.4 ± 1.5 ^e^	0.9872
55 °C																
Dry	615.8 ± 1.0 ^a^	1.3 ± 0.2 ^a^	0.4 ± 0.5 ^a^	0.9994	616.5 ± 1.6 ^a^	1.2 ± 0.1 ^a^	0.3 ± 0.4 ^a^	0.9922	723.5 ± 3.2 ^a^	1.0 ± 0.1 ^a^	0.2 ± 0.1 ^a^	0.9979	599.4 ± 2.6 ^a^	1.0 ± 0.2 ^a^	0.2 ± 0.0 ^a^	0.9970
0.13 *a_w_*	675.9 ± 1.3 ^b^	4.4 ± 0.3 ^b^	0.7 ± 0.2 ^b^	0.9919	631.5 ± 2.1 ^b^	2.7 ± 0.2 ^b^	0.5 ± 0.4 ^b^	0.9904	808.2 ± 1.1 ^b^	2.1 ± 0.1 ^b^	0.4 ± 0.1 ^b^	0.9894	675.0 ± 1.0 ^b^	1.5 ± 0.2 ^b^	0.4 ± 0.1 ^b^	0.9923
0.22 *a_w_*	763.5 ± 2.7 ^c^	5.2 ± 0.1 ^c^	3.1 ± 0.7 ^c^	0.9988	775.5 ± 1.8 ^c^	3.7 ± 0.2 ^c^	1.3 ± 0.7 ^c^	0.9951	881.8 ± 1.9 ^c^	3.3 ± 0.2 ^c^	0.9 ± 0.5 ^c^	0.9867	710.2 ± 1.6 ^c^	3.0 ± 0.1 ^c^	0.7 ± 0.0 ^c^	0.9935
0.33 *a_w_*	1092.7 ± 3.9 ^d^	6.9 ± 0.2 ^d^	4.6 ± 0.9 ^d^	0.9885	865.9 ± 2.6 ^d^	5.9 ± 0.2 ^d^	3.2 ± 0.2 ^d^	0.9807	982.2 ± 2.7 ^d^	4.3 ± 0.1 ^d^	2.0 ± 0.9 ^d^	0.9626	742.9 ± 1.4 ^d^	4.1 ± 0.1 ^d^	1.6 ± 0.3 ^d^	0.9650
0.44 *a_w_*	1201.0 ± 4.2 ^e^	12.5 ± 0.1 ^e^	5.4 ± 1.4 ^e^	0.9895	902.2 ± 1.0 ^e^	12.0 ± 0.2 ^e^	4.4 ± 0.9 ^e^	0.9757	895.9 ± 1.5 ^e^	11.5 ± 0.2 ^e^	3.5 ± 0.7 ^e^	0.9575	892.6 ± 1.4 ^e^	11.4 ± 0.1 ^e^	3.4 ± 0.7 ^e^	0.9872
65 °C																
Dry	748.8 ± 2.3 ^a^	11.6 ± 1.0 ^a^	0.8 ± 0.7 ^a^	0.9994	481.5 ± 1.9 ^a^	7.2 ± 0.1 ^a^	0.7 ± 0.3 ^a^	0.9922	512.1 ± 2.7 ^a^	6.5 ± 0.2 ^a^	0.5 ± 0.7 ^a^	0.9979	482.8 ± 1.7 ^a^	5.5 ± 0.2 ^a^	0.5 ± 0.1 ^a^	0.9970
0.13 *a_w_*	821.7 ± 1.2 ^b^	14.9 ± 0.1 ^b^	1.5 ± 0.8 ^b^	0.9919	509.4 ± 2.7 ^b^	11.6 ± 0.2 ^b^	1.3 ± 0.6 ^b^	0.9904	526.8 ± 1.6 ^b^	9.4 ± 0.1 ^b^	1.1 ± 0.8 ^b^	0.9894	625.9 ± 2.8 ^b^	9.0 ± 0.2 ^b^	1.0 ± 0.7 ^b^	0.9923
0.22 *a_w_*	1293.5 ± 2.7 ^c^	185.0 ± 1.2 ^c^	6.8 ± 0.5 ^c^	0.9988	541.8 ± 1.6 ^c^	62.0 ± 4.9 ^c^	5.0 ± 1.0 ^c^	0.9951	578.2 ± 1.3 ^c^	37.8 ± 1.1 ^c^	2.5 ± 0.3 ^c^	0.9867	631.3 ± 0.8 ^c^	23.5 ± 1.0 ^c^	2.0 ± 0.2 ^c^	0.9935
0.33 *a_w_*	1348.6 ± 3.8 ^d^	206.2 ± 3.1^d^	12.4 ± 2.3 ^d^	0.9885	729.3 ± 3.2 ^d^	141.4 ± 3.2 ^d^	11.8 ± 1.5 ^d^	0.9807	1019.7 ± 3.9 ^d^	96.2 ± 1.3 ^d^	9.5 ± 1.5 ^d^	0.9626	639.71 ± 0.66 ^d^	53.7 ± 2.6 ^d^	6.5 ± 0.9 ^d^	0.9650
0.44 *a_w_*	1495.4 ± 1.7 ^e^	218.1 ± 2.3^e^	17.0 ± 2.1 ^e^	0.9895	1075.3 ± 3.0 ^e^	166.5 ± 3.2 ^e^	13.3 ± 3.1 ^e^	0.9757	1007.8 ± 2.6 ^e^	143.6 ± 3.7 ^e^	10.5 ± 1.9 ^e^	0.9575	754.4 ± 1.6 ^e^	88.8 ± 1.4 ^e^	9.0 ± 0.6 ^e^	0.9872

*: Significant analysis at *p* < 0.05; **: Values are means ± SDs (*n* = 3). Different superscript letters indicate significant differences.

**Table 3:** The literature-derived *S* values for amorphous maltose (M) and maltose/WPI (M-W) mixture with all studied mass ratios at the studied conditions (dry state~0.44 *a_w_* and 45~65 °C).

		M	M-W 7:3	M-W 1:1	M-W 3:7
45 °C	Dry	20.2 ± 4.6 ^a^ *	34.2 ± 3.9 ^a^	36.7 ± 1.5 ^a^	38.0 ± 1.3 ^a^
	0.13 *a_w_*	18.8 ± 0.1 ^b^ **	31.6 ± 0.1 ^b^	34.4 ± 0.1 ^b^	36.3 ± 0.1 ^b^
	0.22 *a_w_*	17.5 ± 0.2 ^c^	30.3 ± 0.1 ^c^	33.8 ± 0.2 ^c^	35.8 ± 0.1 ^c^
	0.33 *a_w_*	16.0 ± 0.1 ^d^	28.8 ± 0.1 ^d^	31.9 ± 0.1 ^d^	33.0 ± 0.1 ^d^
	0.44 *a_w_*	15.5 ± 0.2 ^e^	27.1 ± 0.1 ^d^	29.0 ± 0.1 ^e^	32.3 ± 0.2 ^e^
55 °C	Dry	20.2 ± 4.6 ^a^	34.2 ± 3.9 ^a^	36.7 ± 1.5 ^a^	38.0 ± 1.3 ^a^
	0.13 *a_w_*	19.4 ± 0.1 ^b^	32.9 ± 0.1 ^b^	35.1 ± 0.1 ^b^	36.7 ± 0 ^b^
	0.22 *a_w_*	18.8 ± 0.2 ^c^	32.1 ± 0.1 ^c^	34.3 ± 0.1 ^c^	36.0 ± 0.2 ^c^
	0.33 *a_w_*	18.0 ± 0.1 ^d^	30.8 ± 0.1 ^d^	33.0 ± 0.2 ^d^	35.2 ± 0 ^d^
	0.44 *a_w_*	17.1 ± 0.1 ^e^	29.6 ± 0.3 ^e^	31.8 ± 0.2 ^e^	34.2 ± 0 ^e^
65 °C	Dry	20.2 ± 4.6 ^a^	34.2 ± 3.9 ^a^	36.7 ± 1.5 ^a^	38.0 ± 1.3 ^a^
	0.13 *a_w_*	20.2 ± 0.1^b^	33.7 ± 0.1 ^b^	35.9 ± 0.1 ^b^	37.3 ± 0.1 ^b^
	0.22 *a_w_*	19.9 ± 0.1 ^c^	33.4 ± 0.2 ^c^	35.4 ± 0.1 ^c^	36.9 ± 0.1 ^c^
	0.33 *a_w_*	19.7 ± 0.1 ^d^	32.7 ± 0.2 ^d^	34.8 ± 0.1 ^d^	36.1 ± 0.1 ^d^
	0.44 *a_w_*	19.5 ± 0.1 ^e^	31.7 ± 0.1 ^e^	34.1 ± 0.2 ^e^	35.3 ± 0.0 ^e^

*: Significant analysis at *p* < 0.05; **: Values are means ± SDs (*n* = 3). The literature *S* data were sourced from [23]. Different superscript letters indicate significant differences.

## Data Availability

Data is contained within the article.The data used to support the findings of this study can be made available by the corresponding author upon request.
